# Effectiveness of a lifestyle exercise program for older people receiving a restorative home care service: study protocol for a pragmatic randomised controlled trial

**DOI:** 10.1186/1472-6963-13-419

**Published:** 2013-10-18

**Authors:** Elissa Burton, Gill Lewin, Lindy Clemson, Duncan Boldy

**Affiliations:** 1 Faculty of Health Sciences, Curtin University, Perth, Australia; 2 Research Department, Silver Chain, Perth, Australia; 3 Ageing, Health and Work Research Unit, The University of Sydney, Sydney, Australia; 4 School of Nursing and Midwifery, Curtin University, Perth, Australia

**Keywords:** Physical activity, Exercise, Restorative home care services, Older people, Randomised controlled trial, Study protocol

## Abstract

**Background:**

Restorative home care services help older people maximise their independence using a multi-dimensional approach. They usually include an exercise program designed to improve the older person’s strength, balance and function. The types of programs currently offered require allocation of time during the day to complete specific exercises. This is not how the majority of home care clients prefer to be active and may be one of the reasons that few older people do the exercises regularly and continue the exercises post discharge.

This paper describes the study protocol to test whether a Lifestyle Functional Exercise (LiFE) program: 1) is undertaken more often; 2) is more likely to be continued over the longer term; and, 3) will result in greater functional gains compared to a standard exercise program for older people receiving a restorative home care service.

**Methods/Design:**

Design: A pragmatic randomised controlled trial (RCT) design was employed with two study arms: LiFE program (intervention) and the current exercise program (control).

Setting: Silver Chain, a health and community care organisation in Perth, Western Australia.

Participants: One hundred and fifty restorative home care clients, aged 65 years and older.

Measurements: The primary outcome is a composite measure incorporating balance, strength and mobility. Other outcome measures include: physical functioning, falls efficacy, and levels of disability and functioning.

**Discussion:**

If LiFE is more effective than the current exercise program, the evidence will be presented to the service management accompanied by the recommendation that it be adopted as the generic exercise program to be used within the restorative home care service.

**Trial registration:**

Australian and New Zealand Clinical Trials Registry ACTRN12611000788976.

## Background

As people age there is a tendency to reduce the amount and intensity of physical activity, which can lead to a loss of strength, endurance and balance [1,2]. Such loss of function can then lead to difficulty with everyday living activities and a need for assistance for the older person to remain living in their home. Home care services usually provide this assistance by performing the activity for the older person rather than working with them to assist them to implement strategies that will maximise their function and regain the ability to undertake tasks independently.

In contrast, restorative home care services work with the older person to help them maximise their independence. They do this by incorporating a number of components such as task analysis and redesign, the use of assistive equipment and physical activity programs to improve function, into a short term goal-oriented service [3-5]. In general, restorative home care services provide a “traditional” exercise program, aimed at improving strength and balance to help prevent falls and improve function for the older person. These programs require the older person to complete a set number of exercises, a number of times per day or a number of times per week. The person often completes them for a short period or sporadically, however when they feel they are “better” or have improved their function they often stop.

Recent research has found older people receiving a home care service describe their preferred physical activity as being incidental, such as cleaning the house, walking to the shops and doing the gardening, rather than attending exercise classes or playing sport, which is often preferred by younger generations [6]. Given this and the success of the Lifestyle Functional Exercise (LiFE) program in improving physical function and reducing falls among older people living in the community [7], it was considered important to conduct an RCT to test whether it might be more effective than a traditional exercise program when incorporated into a restorative home care service.

Prior to embarking on the RCT it was necessary to determine whether LiFE was suitable for older restorative home care clients. A pilot study was therefore conducted and LiFE was found to be appropriate for this population [8].

This paper describes the study protocol of the pragmatic randomised controlled trial which compared the effectiveness of LiFE, in both the short and long term, and the current traditional exercise program used in a restorative home care service. It is hypothesised that the LiFE intervention compared to the current exercise program will: 1) be undertaken more often; 2) be more likely to be continued over the longer term; and, 3) result in greater functional gains.

This trial is pragmatic in several aspects including the implementation of the trial within the current restorative home care services of a health and community care organisation in Western Australia. Pragmatic trials are of increasing interest to government health bodies including the National Institute of Health (NIH) and Australia’s Department of Health and Ageing [9], as they provide “real life” results rather than “ideal setting” results often not transferrable to health services.

## Methods

### Study design

This study was a pragmatic, non-blinded, parallel arm randomised controlled trial testing the effectiveness of the LiFE program compared to the current traditional exercise program when delivered within a restorative home care service.

### Participants and setting

The study participants comprised older persons living in Perth suburbs who were referred for a restorative home care service and who met the RCT inclusion criteria. These criteria were: over 65 years of age; assessed by their Care Manager as needing a physical activity program; not having a diagnosis of dementia or other progressive neurological disorders; and, being able to communicate in English.

Silver Chain is an Australian health and community care organisation that delivers a myriad of services, including restorative home care services. The restorative home care services are delivered by an interdisciplinary team consisting of physiotherapists, occupational therapists and registered nurses acting as Care Managers, assisted by aides to provide any direct care needed. Silver Chain delivers two restorative home care services to their older clients; the Home Independence Program (HIP) and the Personal Enablement Program (PEP). HIP is delivered to older people living in the community who need short term assistance to regain their independence, while PEP is delivered to older people who have been in hospital and need the short term service on discharge to help them return to living independently.

HIP and PEP restorative home care services comprise a number of components, including: the promotion of active engagement in daily living activities through work simplification and assistive technology; an exercise program to improve strength, balance and endurance; chronic disease self-management; falls prevention strategies; improvement and maintenance of skin integrity; and medication, continence and nutrition management [10]. HIP is generally delivered for a maximum of 12 weeks and PEP 8 weeks. It was expected that the majority of older people who would be involved in this RCT would be PEP clients, referred after hospital discharge because their referral numbers are higher than HIP.

### Sample size

The sample size was calculated based on 80% power, a 5% significance level and a ‘medium’ effect size of 0.5 [11] related to the two groups, using as the primary outcome, a composite measure incorporating balance, strength and mobility. This effect size was considered sufficient to recommend a change in practice, given that functional improvement is also expected in the current practice group. Based on Peat [12] a sample size of 64 in each group is indicated. Allowing for a 12% drop out rate as found during the pilot study [8], approximately 75 participants in each group were needed, or 150 in total.

### Recruitment, randomisation and allocation concealment

When a client met the eligibility criteria, and agreed that an exercise program would help them achieve their service goals, the Care Manager gave the client: a brief explanation of the study; an introductory letter addressed from the researcher; an information sheet and consent form; and, asked if the researcher could contact them to discuss the project further. If the client agreed, the Care Manager informed the researcher, who called the client within three days to answer any questions and set up a time to visit, gain written consent and complete the baseline data collection. After baseline data were collected, clients were randomised to the intervention or control groups. Notification of group assignment was provided by the researcher to the Care Managers either through email or their computerised phone system and delivery of the allocated exercise program was commenced at the Care Manager’s next visit to that client.

The randomisation process was conducted by a Senior Researcher not involved in the study. Study numbers were randomly allocated to group (‘LiFE’ or ‘current’) using the (simple) random number generator in STATA version 10. Slips of paper with study number and group allocation were then placed in envelopes of the same colour as the paper slip to avoid any tampering with the randomisation. Before sealing the envelopes the study numbers were written on the outside, they were put in sequential order and given to the researcher conducting the trial. The researcher involved in collecting follow up data was not blinded to group allocation.

### Exercise programs

#### Lifestyle and Functional Exercise (LiFE) Program (Intervention)

The LiFE program was developed to improve balance and increase strength in older community dwelling people by embedding exercise into everyday activities [7]. Seven of the exercises in the program are for balance and six are for lower limb strength. It was initially developed as a falls prevention exercise program [13] however there were other outcomes in terms of increased participation and functional improvements that indicate it would be appropriate for our population of restorative home care clients.

After a client had been assessed as needing an exercise program and agreed to participate in the RCT and was randomised to LiFE, the LiFE program was explained and the different exercises described during the Care Manager’s next visit. How these exercises were incorporated into their daily routines, and which they would start with, were discussed and agreed with the older person. The client was also given a manual explaining each of the exercises. Follow up visits were used to monitor how the client was going with the first agreed exercises and to encourage the client to start doing others. In Clemson et al’s original study, LiFE was delivered over five sessions, with two follow up sessions and two phone calls over a six month period [7]. However, in this RCT, clients were expected to be seen every ten to fourteen days by their Care Manager (average three visits), and LiFE was just one aspect of their service that would be discussed during these visits.

#### Current exercise program (Control)

The current exercise program is based on the Otago exercise program developed by Campbell and Robertson [14] which was also initially developed as a falls prevention exercise program. The Otago program has been modified over time by the team delivering restorative home care services, in response to client preferences, to not include weights and, depending on the client’s requirements, sometimes included additional exercises.

After giving written consent and completing baseline data collection, participants allocated to the current exercise program were given a one page instruction sheet (back and front) illustrating the exercises (picture), number of times per day and number of days per week to complete them. The Care Managers explained the exercises they prescribed and during follow up visits they discussed and/or supervised the clients completing new exercises. Based on the Care Managers’ description of “usual” practice, it was expected that an average of three visits involving discussions about their exercises would be included.

**Figure 1:**
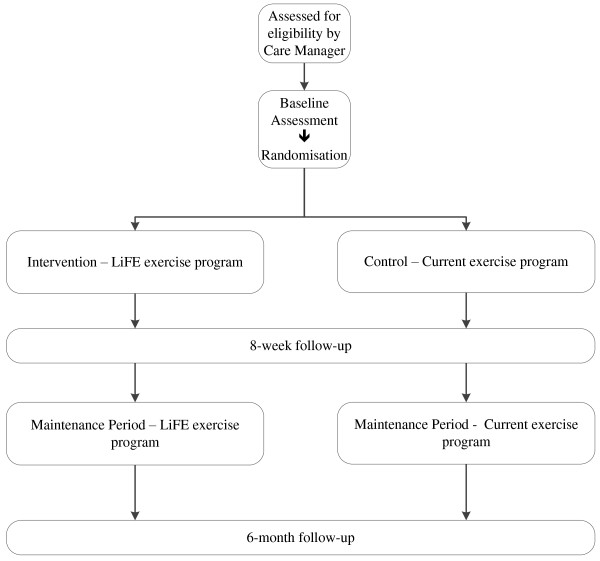
Study schemata.

Depending on group allocation, Care Managers took a LiFE manual or the current exercise program sheet, plus a calendar, to each client at the first visit after randomisation. The clients were asked to tick each day on the calendar they completed the exercises and to keep this until their six month follow up visit. A summary of the study schemata is presented as Figure 1.

#### Data to be collected

All data were collected in the participants’ homes and both activity programs were undertaken either in the participant’s home or different places they may have visited during their week, such as: a shop, park, or footpaths. No specific equipment was needed for either program.

Demographic, home care service and falls information as well as functional data were collected for participants in both the intervention and control groups at baseline, eight weeks and six month follow-up. The six month follow-up occurred four months after the Care Manager ceased the restorative home service with the client. Table 1 provides a basic description of each of the outcome measures that were used in this study.

**Table 1:** Outcome measures and instruments

**Instrument**	**Reference**	**Measures**	**Validity and reliability reported**
Functional Reach	[15]	Balance	Yes
Falls Efficacy Scale	[16]	Fear of falling when completing daily tasks	Yes
Sit-to-stand 1 and 5 times	[17]	Lower body strength and balance	Yes
Timed Up and Go	[18]	Functional mobility	Yes
10-item Vitality Plus Scale	[19]	Potential health-related benefits of exercising	Yes
Tandem walk	[20]	Dynamic balance	Yes
ABC Scale	[21]	Confidence (falls) in completing more challenging tasks	Yes
LLFDI	[22]	Levels of disability	Yes
Disability Component			
LLFDI	[23]	Lower and upper body function	Yes
Function Total			

*Note*. ABC scale is the Activities Specific Balance Confidence Scale, LLFDI the Late Life Function and Disability Instrument.

#### Outcome measures

Mobility, strength and balance (static and dynamic) are all equally important physical attributes for an older person to maintain when living independently. We therefore determined it was more appropriate to calculate a cumulative score of the four physical tests, rather than choose just one, as the primary outcome measure (see Data analysis for more detail).

Secondary outcome measures included: functional reach; falls efficacy scale; sit-to-stand 1 and 5 times; activities specific balance scale; timed up and go; vitality plus scale; tandem walk; the Late Life Function and Disability instruments; and, number of home care services received. These measures (see Table 1) were administered at baseline, eight weeks and six month follow-up. The six month follow-up was important because often exercise programs are not maintained once a service has finished and the Care Manager is no longer there to remind the client of the importance of doing their exercises. This would also show which exercise program, if any, was continued over the longer term.

As described earlier, to determine each client’s exercise frequency they were given a calendar and asked to simply tick each day that they performed the exercises during the trial period. Other data collection techniques for determining compliance were tested during the pilot study [8], such as using clickers twice a week to count the number of times an assigned activity was completed (e.g. Tuesday 10 one leg balance exercises were completed and 15 on Thursday). However, none were found to be successful, whereas getting the client to tick a calendar each day was considered to be more achievable. The number of falls was measured by self-report at baseline and six month follow-up by asking how many falls have you had in the past 6 months. For each individual the study lasted six months, recruitment was expected to be completed within six months and data collection completed twelve months from commencement of recruitment.

### Data analysis

Data will be analysed using the Statistical Package for the Social Sciences computer statistical software, version 19 [24]. Both *intention-to-treat* (ITT) and *per protocol* (PP) analyses will be performed. For each outcome measure, we will calculate the change that has occurred during the intervention period by subtracting the baseline from the 8-week values. Where the distribution of the change is approximately normal, an independent *t*-test will be used to compare the groups. When the data are not normally distributed a Mann-Witney U test will be used. Categorical data will be analysed using a Chi-square test.

The summary variable to be used as the primary outcome measure will be created using clients’ functional reach, chair sit to stand, timed up and go and tandem walk scores. A factor analysis will be carried out to identify the relative importance of these four variables in the dataset, at baseline. This analysis will produce a set of factor loadings or ‘weights’ that can then be used to construct the new summary variable (as a weighted linear combination of the four separate measures above). The weights will be scaled so that, at baseline, the mean of the summary variable is zero and its standard deviation is 1. The same linear combination will be applied to create the summary variable at 8-weeks. Any change in the summary variable over the 8-week period will be assessed for statistical significance in exactly the same manner as the other outcome variables (as described above).

Analyses of the post-testing results at 6 months will consist of a regression model for the outcome of interest, adjusting for the correlations due to the multiple measurements on each individual by treating the person ID as a random effect. Multiple linear regressions will be used to identify any differences between the groups at initial data collection, eight weeks and six months, and to examine differences between the groups in terms of change in measures over the follow up period. A p value of <0.05 will be considered statistically significant. Data analysis will be supervised by a statistician who is not involved in conducting the trial.

### Ethics approval

Ethics approval was obtained from the Curtin University [approval number HR 145/2010] and Silver Chain Human Research Ethics committees [approval number SC-065] prior to the commencement of the study. The RCT was registered with the Australian and New Zealand Clinical Trials Registry ACTRN12611000788976.

## Discussion

Population ageing is one of the major 21^st^ century challenges facing many countries around the world. Substantially increased numbers of older people and decreasing proportions in the workforce, together mean that services to assist older people to maximise their health and wellbeing and remain living independently in the community will become increasingly more essential. Restorative home care services are short-term and have been shown to reduce the need and costs for on-going services in this older population [25]. Ensuring that these services are maximally effective is crucial. Strategies to encourage individuals to increase their activity levels and overall physical fitness are an essential aspect of these restorative home care services. We need to identify which strategies work best with this target population.

In this paper we have described our protocol for testing the effectiveness of a lifestyle exercise program compared to the current exercise program used in a restorative home care service. This protocol may assist other health services researchers wanting to conduct a pragmatic trial to compare the effectiveness of a new/different service component with a current component while continuing to deliver the service to its clients.

Health care services/researchers may also choose similar outcome measures as those used for this study, as they are all validated and reliable tools and have been used by others investigating older people receiving long term home care services [26]. Other researchers or health care organisations may find the summary variable, or principles used to create it, useful because living independently requires an improvement across many physical domains not just one.

The study is being conducted in an existing restorative home care service operating within a health and community care organisation. The results should be generalisable to other similar services. Engagement with a health care organisation is a continuum and this trial is an example of a research-operational partnership where research is motivated by a desire by all parties to increase both service and cost effectiveness.

## Abbreviations

LiFE: Lifestyle and functional exercise program; HIP: Home independence program; PEP: Personal enablement program; ITT: Intention-to-treat; PP: Per protocol.

## Competing interests

The authors declare that they have no competing interests.

## Authors’ contributions

EB: participated in the study concept and design, acquisition of data, analysis and interpretation of data and drafting of the manuscript. GL: participated in the study concept and design, interpretation of data and critical revision of the manuscript. LC: was part of the investigation team who developed the LiFE exercise program, trained the Care Managers to deliver LiFE, provided input into the study design and provided critical revision of the manuscript. DB: participated in the study design and provided critical revision of the manuscript. All authors read and approved the final manuscript.

## Authors’ information

The data used in this article will form part of a doctoral thesis and the first author (EB) would ask permission to present this article as part of her PhD thesis.

## Pre-publication history

The pre-publication history for this paper can be accessed here:

http://www.biomedcentral.com/1472-6963/13/419/prepub
